# Label‐Free High‐Throughput Leukemia Detection by Holographic Microscopy

**DOI:** 10.1002/advs.201800761

**Published:** 2018-10-11

**Authors:** Matthias Ugele, Markus Weniger, Manfred Stanzel, Michael Bassler, Stefan W. Krause, Oliver Friedrich, Oliver Hayden, Lukas Richter

**Affiliations:** ^1^ In‐Vitro DX and Bioscience Department of Strategy and Innovation Siemens Healthcare GmbH Günther‐Scharowsky‐Str. 1 91058 Erlangen Germany; ^2^ Department of Chemical and Biological Engineering Institute of Medical Biotechnology Friedrich‐Alexander‐University Erlangen‐Nuremberg Paul‐Gordan‐Str. 3 91052 Erlangen Germany; ^3^ Analysesysteme und Sensorik Fraunhofer IMM Carl‐Zeiss‐Str. 18‐20 55129 Mainz Germany; ^4^ Medizinische Klinik 5 Hämatologie and Internistische Onkologie Ulmenweg 18 91054 Erlangen Germany; ^5^ Heinz‐Nixdorf‐Chair of Biomedical Electronics Department of Electrical and Computer Engineering TranslaTUM Campus Klinikum rechts der Isar Technical University of Munich Ismaningerstr. 22 81675 Munich Germany

**Keywords:** digital holographic microscopy, label‐free detection, leukemia, microfluidics

## Abstract

Complete blood count and differentiation of leukocytes (DIFF) belong to the most frequently performed laboratory diagnostic tests. Here, a flow cytometry‐based method for label‐free DIFF of untouched leukocytes by digital holographic microscopy on the rich phase contrast of peripheral leukocyte images, using highly controlled 2D hydrodynamic focusing conditions is reported. Principal component analysis of morphological characteristics of the reconstructed images allows classification of nine leukocyte types, in addition to different types of leukemia and demonstrates disappearance of acute myeloid leukemia cells in remission. To exclude confounding effects, the classification strategy is tested by the analysis of 20 blinded clinical samples. Here, 70% of the specimens are correctly classified with further 20% classifications close to a correct diagnosis. Taken together, the findings indicate a broad clinical applicability of the cytometry method for automated and reagent‐free diagnosis of hematological disorders.

## 1 Introduction

Routine diagnosis of hematological disorders requires stained blood smear analysis and remains the gold standard. Modern automated hematology analyzers usually provide a five‐part differential separating neutrophils, basophils, eosinophils, monocytes, and lymphocytes, but only flag atypical leukocytes as “abnormal,” which provides no clear classification due to method limitations.^1^ To accurately interpret peripheral blood smears, well‐stained samples and time‐consuming microscopic analyses are required, which still show high interobserver variation.^2^, ^3^ Additional methods, like flow cytometry, rely on sometimes tedious sample preparation and labeling for the classification of benign and malignant leukocytes, which is expensive and time‐consuming. To overcome the analytical limitations of today's methods, the analysis of native blood cells in suspension would be highly attractive for routine clinical diagnosis. Digital holographic (DH) microscopy was suggested as method of choice for label‐free cell imaging, because the phase contrast provides rich intracellular information due to subtle refractive index changes at internal structures. Most work discussed the analysis of cancer cells,^4^, ^5^, ^6^, ^7^, ^8^, ^9^ red blood cells,^10^, ^11^, ^12^, ^13^, ^14^, ^15^ point‐of‐care applications,^16^, ^17^ and leukocyte differentiation with cells resting on surfaces.^11^, ^18^, ^19^ In particular, machine learning has been used for cellular classification, demonstrating the potential of these approaches in combination with DH. Various machine learning algorithms were compared for the differentiation of erythrocyte morphologies, such as discocytes, echinocytes, and spherocytes, and for the label‐free detection of malaria‐infected red blood cells by digital in‐line holographic microscopy.^20^, ^21^ Holographic microscopy and deep learning were combined by Jo et al. for the screening of anthrax spores with subgenus specificity.^22^ In addition, Pavillon et al. report the label‐free detection of macrophages activated by lipopolysaccharide by a combination of DH, Raman spectroscopy and machine learning.^23^ Yoon et al. demonstrated the identification of B, CD4+ T, and CD8+ T cells with an overall accuracy of over 75% by optical diffraction tomography and machine learning.^24^ Recently, imaging results of leukocytes in flow conditions were reported, which were still inferior to today's high‐throughput scatter analysis methods in hematology analyzers.^25^, ^26^ More resolution power of DH is required to demonstrate clinically relevant information. Leukemia is responsible for more than 67 000 deaths per year in the EU and USA.^27^, ^28^ Therefore, simple and fast, automated label‐free leukemia detection would be a significant advancement of the field to match the unmet need of clinical routine diagnosis. Among other, automated hematology analysis of such complex diseases would potentially reduce manual differentiation of leukocytes (DIFF) efforts in central laboratories and could improve patient diagnosis at the point‐of‐care level.

To match such clinical needs, we adopted a differential digital holographic microscope with a depth of field (DOF) of ±2.3 µm, which is the half width of the full depth of field range,^29^ centered in a 2D hydrodynamically focused sample stream of a microfluidic channel. The DOF was chosen to achieve a balance between spatial resolution (0.6 µm (Rayleigh)) and sufficient phase information from the contrast‐rich, but randomly orientated nuclei in flow, since already small changes to greater/smaller DOF would lead to a loss of contrast. Furthermore, the DOF around the focal plane ensured that at least an ≈1/3 sub‐monolayer of the heterogeneous leukocytes, with cell diameters ranging typically from 6 to 12 µm,^30^ are in focus, irrespective of minor variabilities (≤1 µm) of the leukocyte position in the sample stream. With these flow and imaging conditions, we were able to discriminate the granulocyte types and to perform a five‐part DIFF with healthy donor samples, without any requirement for extensive sample preparation, leukocyte staining, and additional autofocusing effort. In addition, the high phase contrast of untouched cells close to in vivo conditions even allowed a discrimination of clinical leukemia samples. Based on principal component analysis (PCA) and morphological parameters using training data sets of reconstructed, native leukemic cells, we developed a gating strategy for the differentiation of nine leukocyte subtypes, which enabled us to extend the DIFF to pathological samples, such as acute myeloid leukemia (AML), acute lymphocytic leukemia (ALL), chronic lymphocytic leukemia (CLL), and myeloproliferative neoplasms (MPN).

## 2 Results

### 2.1 Label‐Free Five‐Part DIFF of Leukocytes

**Figure 1:**
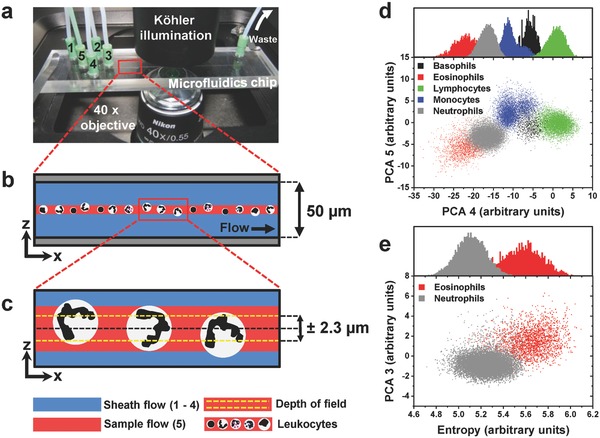
Microfluidics cell presentation and five‐part DIFF of leukocytes. a) Four sheath inlets (indicated as 1–4) form a 2D sheath flow around the sample (inlet 5). b) Schematic side view of the microfluidics channel shown in (a). A focused monolayer of enriched leukocytes is formed. c) Enlarged schematics of the sample flow shown in (b). The physical DOF of ±2.3 µm results in imaging of cell segments which pass the optical field in random positions. d) Purified leukocyte populations of multiple healthy donors separated by PCA. e) The combination of PCA component and entropy significantly improved the separation efficiency of eosinophils and neutrophils.

We chose a customized differential holographic microscope (Ovizio Imaging Systems), comprising a 528 nm super‐LED Koehler illumination and a 40× objective (NA 0.55, Nikon), for high‐throughput (105 fps, acquisition time 5 µs), label‐free in‐flow imaging of leukocytes (**Figure** **1**a and Figure S1, Supporting Information). The resulting depth of field of ±2.3 µm required precise focusing of cells, which was ensured by 2D hydrodynamic focusing of the sample in a polymethyl methacrylate (PMMA) microfluidics chip with a channel height of 50 µm, a width of 500 µm, and four sheath inlets (Figure 1a,b). We used a six‐port injection valve with a scalable, noncalibrated sample loop, which allowed relative counting of cell populations to enable a measurement of a high dynamic range of leukocyte concentrations (1–200 × 10^3^ cells µL^−1^) under identical flow conditions. The 2D sheath flow was favored to avoid any contacts of leukocytes with the channel walls, which could possibly lead to activation of cells or apoptosis and thus, could bias the label‐free differentiation of leukocytes. With a sample flow rate of 0.024 µL s^−1^, the sample stream height was controlled at 8 µm, which corresponds to an average leukocyte diameter, and allowed us to reproducibly image sub‐monolayers of untouched leukocytes with varying diameters within <3 h after blood drawing (Figure 1c and Figure S2g,h, Supporting Information). We assumed to have more comparable contrast between the leukocyte types with a fixed focal height as compared to imaging of sedimented leukocytes on a glass slide's surface. On surfaces, the large diameter differences require a continuous adjustment of focal height, which is not compatible with a parallelized high‐throughput workflow.

To test the applicability of our setup for the main leukocyte types, we first examined the label‐free five‐part DIFF of highly enriched basophils, eosinophils, lymphocytes, monocytes, and neutrophils with population purities of 91–99% from multiple healthy donors (Figure S2i–o, Supporting Information). For the calibration, we only used perfectly focused cells segmented out of reconstructed phase images (Figure S4a, Supporting Information) and split the data set into training (75%) and test (25%) data (*n*_total_ = 66 554). We trained a support vector machine (SVM, radial kernel) on the training data and received an overall accuracy of 92% on the test data set, with sensitivities ≥80% for each of the five leukocyte types (basophils = 80%, eosinophils = 89%, lymphocytes = 89%, monocytes = 91%, neutrophils = 95%; Figure S3a, Supporting Information). The dot plots in Figure 1d show a high separation of basophil, lymphocyte, monocyte, and neutrophil populations by PCA on calculated morphological parameters (Table S1, Supporting Information). Overlapping clusters of eosinophil and neutrophil populations could be further discriminated with a combination of PCA and morphological values, such as entropy (Figure 1e). We observed that the use of a DH setup with smaller/larger DOF than ±2.3 µm (DOF of ±1.9 µm (λ = 445 nm), DOF ± 2.8 µm (λ = 630)) would lead to a loss of information about the inner structure of the leukocytes, due to averaging or cell orientation effects of the nucleus, and therefore lead to a decreased overall classification accuracy by SVM (data not shown). For that reason, we concluded that a DOF of ±2.3 µm was preferably for leukocyte differentiation.

### 2.2 Differentiation of Healthy and Leukemic Samples

To assess whether our system was also suitable for the differentiation of leukocyte fractions without prior enrichment of subtypes, we directly isolated native leukocytes from whole blood and derived a five‐part DIFF gating strategy based on the differentiation of highly enriched leukocyte subtypes and clustering of multiple healthy samples (Figure S4b, Supporting Information). We managed to image cells with minimum sample preparation within a time frame of <3 h from blood drawing to DH imaging, in order to exclude interferences from potentially in vitro activated cells (Figure S2a–f, Supporting Information). Selective lysis of erythrocytes was followed by depletion of erythrocyte fragments and the remaining leukocytes were resuspended in plasma without any labeling or fixation. The results obtained from ten healthy donors were compared with the corresponding data of an ADVIA2120 hematology system. Neutrophils (*R* ^2^ = 0.97), lymphocytes (*R* ^2^ = 0.95), and eosinophils (*R* ^2^ = 0.89) showed high correlation, whereas monocytes showed considerably lower correlation (*R* ^2^ = 0.56) to the ADVIA2120 data (Figure S4d–g, Supporting Information). This inconsistency may be due to the additional LUC (large unstained cells) population displayed by the ADVIA2120,^31^, ^32^ which is supported by the observed increase in the correlation to *R* ^2^ = 0.89 by merging monocyte and LUC populations (Figure S4h, Supporting Information). Basophils showed no correlation (Figure S4i, Supporting Information).

**Figure 2:**
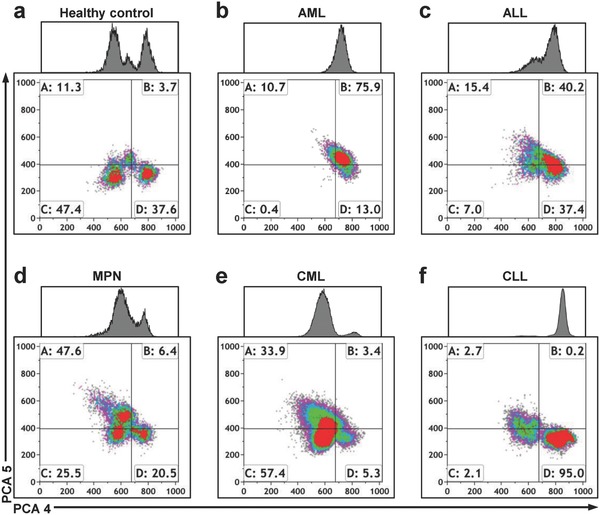
Label‐free, ungated single cell analysis allows leukemia subtyping. a) Label‐free, ungated density plots of healthy control and b) samples with acute myeloid leukemia (AML), c) acute lymphocytic leukemia (ALL), d) myeloproliferative neoplasm (MPN), e) special MPN chronic myelogenous leukemia (CML), and f) chronic lymphocytic leukemia (CLL). Parameters PCA4 and PCA5 are plotted. Each density plot shows representative single cell data from one sample. Percentages of cells are indicated for each plot quadrant.

Encouraged by the five‐part DIFF robustness of our system, we analyzed clinical samples with AML (*n* = 10), ALL (*n* = 1), CLL (*n* = 2), and MPN, including osteomyelofibrosis (OMF, *n* = 2), *polycythemia vera* (PV, *n* = 1), post essential thrombocythemia (ET, *n* = 1), chronic myelogenous leukemia (CML, *n* = 1), chronic myelomonocytic leukemia (CMML, (MPN/MDS), *n* = 1), and MPN NOS (not otherwise specified, *n* = 1) (Table S2, Supporting Information). To investigate whether healthy and leukemic samples could already be differentiated by four‐quadrant gating of raw leukocyte data, we first determined the distribution of cells from healthy donors in quadrants A–D (**Figure** **2**a and Figure S5a–j, Supporting Information). We found that all leukemic samples were disparate from the healthy distributions of 7–30% in A, 2–7% in B, 32–62% in C, and 10–43% in D, in at least one quadrant (Figure S5k, Supporting Information). These findings show that leukemic samples were clearly detected by our system, which provided the basis for further investigations. Interestingly, ungated raw leukocyte data did not only reveal leukemic samples, but also allowed distinction of different leukemias. Subtyping of AML and MPN samples was feasible in some particular cases, for example, CML/CMML, as well as the differentiation of ALL and CLL samples from AML and MPN (Figure 2b–f and Figure S6, Supporting Information). In summary, our data indicate that a separation of AML, ALL, CLL, and CML/CMML is achievable without a complex gating strategy of the leukocyte data.

### 2.3 Gating Strategy for Leukemia Typing

**Figure 3:**
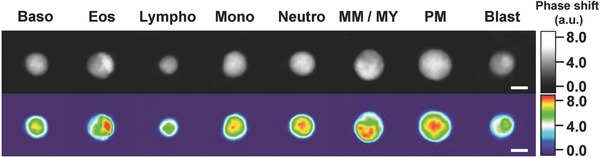
Label‐free DIFF of eight leukocyte subtypes. Top row: reconstructed phase images of leukocyte subtypes. Bottom row: corresponding heat maps of top row phase images. Single cell data of multiple healthy and leukemic samples were used for the differentiation of leukocyte subtypes. Hematology analyzer data of healthy and blood smear analysis of leukemic samples were used as reference. Baso, basophil; Eos, eosinophil; Lympho, lymphocyte; Mono, monocyte; Neutro, neutrophil; MM/MY, meta‐/myelocyte; PM, promyelocyte. Scale bars are 5 µm.

**Figure 4:**
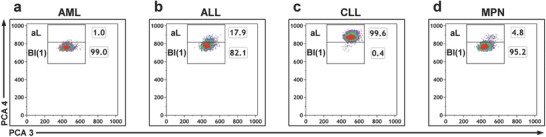
Differentiation of ALL and CLL from AML and MPN. b) Acute lymphocytic leukemia (ALL) and c) chronic lymphocytic leukemia (CLL) are differentiated from a) acute myeloid leukemia (AML) and d) myeloproliferative neoplasm (MPN) by characteristic distribution of atypical lymphocytes (aL) and blasts (Bl(1)). Density plots show representative data of single samples. PCA components are plotted. Percentages of cells are indicated for each gate in the dot plots.

To ensure a conclusive and reliable differentiation especially of AML and MPN samples, we developed a gating strategy based on the five‐part DIFF results of healthy samples, which allowed distinct subtyping of leukemias by different distributions of leukocyte subtypes. At first, we tried to differentiate leukocyte subtypes beyond the five‐part DIFF, and used the blood smear data from leukemic samples as reference for the presence or absence of malign cell types, such as immature granulocytes and blasts. Overall, we successfully differentiated eight different leukocyte subtypes, including basophils, eosinophils, lymphocytes, monocytes, neutrophils, promyelocytes, meta‐/myelocytes and blasts, with a combination of PCA parameters, and five additional morphological parameters (entropy, energy, homogeneity, optical height maximum, and sphericity, see Figure S7 and Table S1, Supporting Information). Metamyelocytes and myelocytes could not be further separated due to ambiguity, but may be resolvable with more clinical samples (Table S2, Supporting Information). To verify the discrimination strategy, we compared the morphological shape of leukocytes in the assigned gates and observed significant morphological differences between all identified cell types, which strongly supports our classification strategy (**Figure** **3**). A ninth cell type, which we named “atypical lymphocytes,” was clearly identified by our gating strategy (**Figure** **4**c and Figure S7h, Supporting Information) but did not show any significant morphological differences to lymphocytes.

Next, we investigated the applicability of our gating strategy for the differentiation of ALL, CLL, AML, and MPN samples. Therefore, we reviewed the differences in the percentage distribution of lymphocytes and atypical lymphocytes. We found that ALL and CLL samples were differentiable from AML and MPN samples by their amount of lymphocytes and atypical lymphocytes (Figure 4 and Table S3, Supporting Information).

**Figure 5:**
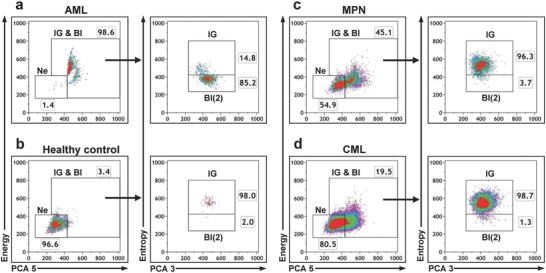
Gating strategy to differentiate AML and MPN. a) Gating strategy to differentiate acute myeloid leukemia (AML) from c) myeloproliferative neoplasm (MPN) and b) healthy control by characteristic distribution of neutrophils (Ne), immature granulocytes (IG), and blasts (Bl(2)). d) Chronic myelogenous leukemia (CML) is differentiated from other MPN by a unique scatter pattern. Gate IG & Bl = immature granulocytes and blasts. Density plots show PCA parameters and a combination of PCA and energy/entropy. Each dot plot shows representative data from a single sample. Percentages of cells are indicated for each gate.

For the differentiation of AML and MPN samples, we examined the differences in the percentage distribution of neutrophils, blasts, and immature granulocytes. All AML samples showed <25% neutrophils in combination with >5% blasts (**Figure** **5**a and Table S3, Supporting Information). In comparison, MPN samples displayed >25% neutrophils in combination with >10% immature granulocytes (Figure 5c and Table S3, Supporting Information). All AML and MPN samples were clearly distinguishable by these characteristic patterns. Moreover, CML/CMML samples were differentiable from other MPN samples by their neutrophil amount of >55% (Figure 5d and Table S3, Supporting Information). Overall, the comparison of the distributions of atypical lymphocytes, lymphocytes neutrophils, blasts, and immature granulocytes enabled us to differentiate ALL, CLL, AML, and MPN samples.

### 2.4 Comparison of AML from Diagnosis and Remission

**Figure 6:**
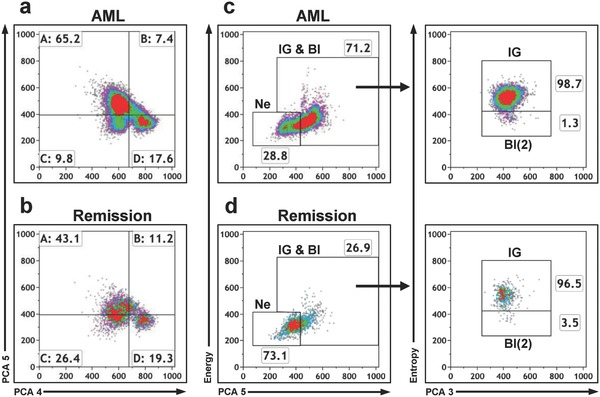
Progression of AML from diagnosis to remission. a,b) Ungated density plots of acute myeloid leukemia (AML) directly after first diagnosis and same patient in remission. c,d) Percentage distribution of neutrophils (Ne), immature granulocytes (IG), and blasts (Bl(2)) from diagnosis to remission. Gate IG & Bl = immature granulocytes and blasts. PCA parameters and a combination of PCA and morphological parameters energy/entropy are plotted. Percentages of cells are indicated for each gate or density plot quadrant.

To track the pattern of native leukocytes during the course of therapy, we analyzed two samples from one AML patient to reveal cancer remission after chemotherapy. The first sample was measured directly after diagnosis and the second at day 47 after chemotherapy in remission. We observed an increase from 13.6 to 36.6% neutrophils and a decrease from 37.1 to 14.4% immature granulocytes in remission (**Figure** **6** and Table S3, Supporting Information). Consequently, the remission sample was neither classified as healthy but rather as MPN, because of the presence of immature granulocytes.

### 2.5 Analysis of Blinded Clinical Samples

Finally, we analyzed 20 blinded clinical samples to investigate the reliability of our leukemia differentiation strategy based on a limited data set of pathological samples. Overall, 14 of 20 samples were in complete accordance to clinical diagnosis and in four samples, a morphologically closely related diagnosis was found by DH (Table S4, Supporting Information). One sample was interpreted as healthy instead as ALL and one was assigned MPN, although it was a patient in complete remission of his CML with a normal differential count. Inaccuracies in the assignment of leukemic samples occurred with the differentiation of monocytic AML samples with lower blast amount and MPN samples. Here, two monocytic AML samples (blinded sample 1 and 9) were incorrectly assigned as MPN. Additionally, MPN samples were not correctly recognized in all cases. Blinded sample 12, an MPN with increased numbers of myeloid blasts, monocytes, and red cell precursors, was assigned AML. Since the amount of ALL and CLL reference samples was low (*n* (ALL) = 1, *n* (CLL) = 2), certain differentiation of ALL and CLL was not possible and samples with >40% lymphocytes in combination with >3% atypical lymphocytes were assigned as lymphatic leukemia. All CLL samples were correctly classified as lymphatic leukemias by this scheme. Taken together, we could show on blinded samples that our new method is able to discriminate hematological disorders.

## 3 Discussion and Conclusion

We report a label‐free nine‐part DIFF technique of native leukocytes by high‐throughput digital holographic microscopy, which enabled us to (1) discriminate healthy and pathological samples and (2) classify leukemias, as AML, lymphatic leukemia, and MPN in clinical samples. We conclude that in‐flow imaging using a fixed focal height and moderate DOF to image cell sections is more favorable than lens‐free holography.^11^, ^18^, ^25^ At the same time, the robust workflow does not require refocusing efforts to image sedimented leukocytes with variable diameters,^11^, ^18^ which reduces the complexity of future readers. One of the major advantages of DH is the possibility of postimaging autofocusing. Autofocusing procedures in DH are well studied and numerous autofocusing criteria have been demonstrated for different holographic imaging applications and were successfully used to image various samples.^33^, ^34^, ^35^, ^36^, ^37^, ^38^, ^39^ In addition, Wu et al. proposed the use of use of machine learning for autofocusing as an alternative to conventional approaches.^40^ However, autofocusing procedures are still time‐consuming and therefore are not compatible with a high‐throughput workflow in a clinical environment.

In parallel to the analysis of phase images, we also looked into intensity images of different leukocyte types, to identify the most relevant DH parameters for leukocyte discrimination, since parameters as cell area or equivalent diameter can also be obtained independently from DH. It was observed that intensity images of leukocytes did not show enough morphological differences to be discriminated from each other, with the exception of eosinophils (see Figure S8, Supporting Information). For that reason we did not make any further efforts to achieve a discrimination of leukocytes with the amplitude information only and concluded that the parameters based on phase values, for example, optical volume, optical height maximum, contrast, and energy were most relevant for the discrimination of leukocytes using the customized Ovizio microscope.

The results obtained from SVM classification and PCA visualization indicated that the random orientation of leukocytes and thus, the orientation of the nucleus as major contributor to the phase contrast, did not substantially affect the subtyping of the main five leukocyte types. As a control, we examined the cell morphology in the separated cell populations. The apparent conformity observed within the individual populations supported our assumptions (Figure S3b, Supporting Information) and verifies that label‐ and sample preparation‐free five‐part DIFF of leukocytes can be performed without the need for continuous optical calibration. In addition, the accordance of the DH five‐part differential results with the ADVIA2120 proved the stability of our system, although basophils showed no correlation, as expected, when considering that basophil correlation is also weak among common analyzers.^31^, ^41^ In contrast to state‐of‐the‐art hematology analyzers, which mainly provide flags in the case of abnormal cell morphologies,^31^, ^42^ we demonstrated the potential of this method for automated leukemia detection and classification. Our results indicate the possibility for monitoring of leukemia progression and the analysis of blinded samples proved the feasibility of our strategy. For a refinement of our classification strategy, reference samples will be extended and manual gating will be optimized by the use of clustering algorithms, as already demonstrated for mass‐ and flow cytometry data.^43^ After optimization, our approach bears the potential to circumvent the interobserver dependence of peripheral blood smear analysis, which is a known variability in the diagnosis of hematological disorders.^3^, ^44^, ^45^, ^46^ Finally, we envision that leukocyte viability and function close to in vivo conditions^47^ potentially add promising new information to hematology diagnosis, which is not covered by today's CBCs measured using automated hematology analyzer.

## 4 Experimental Section

*DH Microscopy*: Digital holographic microscopy was performed with a customized differential holographic microscope provided by Ovizio Imaging systems, Belgium, which uses patented “differential digital holographic microscopy.”^48^, ^49^ The microscope comprised a partially coherent triggered 528 nm Oslon PowerStar SLED (Osram) Koehler illumination unit in transmission mode and a 40× NA 0.55 Nikon CFI LWD 40×Cremove objective. By using a low‐coherent light source, the image degradation caused by the noise of a coherent laser is eliminated and the quality of the image is improved.^50^, ^51^, ^52^ The light beam emitted from the LED first passes the sample, which is located in the back focal plane of the microscope objective. The light beam is then split by a diffraction grating into a diffracted beam (reference) and a nondiffracted beam (object beam). The diffraction beam is then recombined with the object beam and focused on an imaging device (see Figure S1, Supporting Information). A detailed description of the microscopic setup and working principle is described in ref. 48, 49. The used light source and objective combination resulted in an optical depth of field of ±2.3 µm, which is the half width of the full depth of field range,^29^ and a lateral resolution of 0.6 µm (Rayleigh). A PointGrey Grashopper GS3‐U3‐32S4 camera was used for high‐throughput (105 fps, acquisition time 5 µs) imaging of leukocytes. Reconstruction of phase images from recorded holograms was performed by Poisson integration using the commercially OsOne Software Version 5.1 (Ovizio Imaging Systems, Belgium).

*Microfluidics*: Microfluidics PMMA chips with a channel height of 50 µm, a width of 500 µm, a total length of 5000 µm, and five inlets were purchased from Fraunhofer ICT‐IMM, Mainz, Germany. A neMESYS Base120 pump system with five modules (cetoni GmbH) was equipped with 2.5 mL gas tight syringes (VWR) and used for focusing of cells in the microfluidics setup. Sheath flow conditions were established using 0.9% polyvinylpyrrolidone (PVP, average mol. wt. 1.3 MDa, Sigma‐Aldrich) in autoMACS Rinsing Solution, Milteny Biotec (PBS, 2 × 10^−3^ m EDTA, pH 7.2) as sheath flow buffer. autoMACS Rinsing Solution was used as sample flow buffer. A total flow rate of 0.398 µL s^−1^ was used for 2D hydrodynamic focusing of cells with a sample flow rate of 0.024 µL s^−1^, an *x*/*y*‐sheath flow rate of 0.037 µL s^−1^, an upper *z*‐sheath flow rate of 0.1 µL s^−1^, and a lower *z*‐sheath flow rate of 0.2 µL s^−1^. A six‐port injection valve (V‐451 Injection Valve Bulkhead 2 Position‐6‐Port .040 Black, IDEX Health & Science) was used for in‐flow sample injection. Microfluidics components (tubes, connections) were purchased from IDEX Health & Science.

*Determination of Sample Flow Height*: 0.1 m methylene blue in autoMACS Rinsing Solution was used to examine the sample stream height at different sample flow conditions, using a Leica DM 2500 M microscope equipped with a Baumer HXG20 camera. 2D sheath flow conditions remained constant, as described above, and the sample flow containing 0.1 m methylene blue was varied from 1 to 0 µL s^−1^ (1, 0.8, 0.6, 0.4, 0.2, 0.1, 0.08, 0.06, 0.04, 0.02, 0.01, and 0 µL s^−1^). The gray value for each sample flow condition was quantified at three different positions inside the channel using ImageJ. For each flow condition three measurements were performed. For the preferred sample flow of 0.024 µL s^−1^, a sample flow height of 8 µm was measured (Figure S2g,h, Supporting Information). This method was preferably used instead of particle tracking53 or light scattering approaches,^13^ which are highly applicable for analyzing the position of objects in microfluidics flows, but are unsuitable to absolutely quantify the sample stream height in the present microfluidics system used for 2D focusing with multiple sheath flows.

*Data Processing and Analysis*: The floating point phase shift pixel values for each recorded phase image within the interval [0, 8] were converted to grayscales. On the resulting grayscale images, a background image was calculated by determining the pixel‐by‐pixel median gray value from the first 11 images. This background image was subtracted from each phase grayscale image for background correction. For each corrected image, a binary picture was generated by thresholding at the gray level of 28. Resulting holes in binary images were removed. As a single image in general contained more than one cell, all object contours were segmented out of the resulting images. Afterward, parameters for each object were calculated out of the segmented contour, based on the pixel values inside the contour and based on the gray level co‐occurrence matrix (for details of calculated features, see Table S1, Supporting Information).^54^ The resulting segmented object parameters were filtered to eliminate artifacts, platelets, and cells out of focus (see Figure S4a, Supporting Information). The following parameter intervals were used to remove invalid segmentations: radius variance < 0.2, >1.2; biconcavity < −0.3, >0.2; aspect ratio > 1.25; cell area < 20, >300; optical height minimum < 0.9; solidity < 0.95; contrast < 2; equivalent diameter < 6.9; optical height maximum > 3.8; circularity < 0.84; sphericity < 0.35; and mass center shift > 2.5. The resulting segmentations were assumed to be valid, focused cells. For five‐part differential of purified leukocyte populations (basophils, eosinophils, lymphocytes, monocytes, and neutrophils) the R package caret was used.^55^ The dataset (*n*_total_ = 66 554) was split into training (75%) and test dataset (25%). The training data set class imbalance was corrected by common upsampling techniques that matched the number of the five leukocyte types to the same level. A support vector machine with radial kernel was trained on the training dataset; the SVM was then used to predict the leukocyte populations on the test data set. The statistical significance of the morphological parameters between cell types was checked using ANOVA in the context of SVM for leukocyte five‐part differential. All of the used features had an ANOVA *p*‐value below 0.01, for that reason all features were included in the analysis. PCA on morphological parameters was performed on the complete dataset with all five leukocyte subtypes (principal components PCA 1–3) and on basophil and lymphocyte subsets only (principal components PCA 4–6; see Table S1, Supporting Information). The gating strategies for five‐ and nine‐part DIFF of leukocytes were developed using Kaluza flow cytometry analysis, version 1.2 (Beckman Coulter). Hematology analyzer data of healthy and blood smear analysis of clinical samples were used as reference for the presence/absence of leukocyte subtypes.

*Human Samples*: Leukocytes for reference measurements were isolated from healthy donors. Clinical samples were obtained from the Medizinische Klinik 5, Hämatologie and Internistische Onkologie, Erlangen, Germany. All human samples were collected with informed consent and procedures approved by application 316_14B (healthy donors) or 194_15B (clinical samples) of the Ethikkommission der Universität Erlangen.

*Purification of Leukocyte Subpopulations*: T‐cells, eosinophils, and neutrophils for five‐part differential reference measurements were purified from EDTA coagulation inhibited peripheral blood by immunomagnetic depletion using Miltenyi Biotec MACSxpress Pan T‐Cell Isolation Kit (130‐098‐193), Miltenyi Biotec MACSxpress Eosinophil Isolation Kit (130‐104‐446), or Miltenyi Biotec MACSxpress Neutrophil Isolation Kit human (130‐104‐434). Monocytes and basophiles were isolated from PBMCs (peripheral blood mononuclear cells) isolated by Ficoll gradient or by using the Miltenyi Biotec MACSprep PBMC Isolation Kit (130‐115‐169) and purified by subsequent immunomagnetic depletion using Miltenyi Biotec Pan Monocyte Isolation Kit (130‐092‐537) or Miltenyi Biotec Basophil Isolation Kit (130‐092‐662). Whenever needed, remaining red blood cells were removed using the Miltenyi Biotec MACSxpress Erythrocyte Depletion Kit (130‐098‐196). Purity of the different cell preparations was quantified by FACS using a BD Acurri C6 flow cytometer. Leukocytes were fluorescently labeled with anti‐CD123‐FITC (basophils), anti‐CD16‐FITC and anti‐CD66‐PE (eosinophils), anti‐CD3‐FITC (T‐cells), anti‐CD14‐PE (monocytes), and anti‐CD16‐FITC (neutrophils). Remaining cell debris and platelets in the purified leukocyte populations were excluded by carefully adjusting the FACS threshold settings prior to the calculation of the leukocyte population purity. The purified cell populations were in the range of 91–99% (basophils 92–95%, eosinophils 92–95%, monocytes 91–93%, neutrophils 95–99%, T‐cells 94–98%).

*Isolation of Leukocytes Fractions from Whole Blood*: Blood samples were processed within a time frame of maximum 3 h from drawing blood to DH imaging. Leukocytes from healthy and clinical samples were isolated by selective hypotonic water lysis of erythrocytes as previously described.^47^ For healthy samples, 1 mL whole blood, and for clinical samples, 0.5–1 mL whole blood was processed. Remaining erythrocyte fragments, which could possibly interfere with the imaging of leukocytes, were removed using the Miltenyi Biotec MACSxpress Erythrocyte Depletion Kit (130‐098‐196). After spinning at 400 g for 10 min to diminish the volume of the erythrocyte free cell suspension, the remaining pellet was resuspended in plasma without any labeling or fixation.

*Blood Smear and ADVIA2120 Reference Measurements*: Peripheral blood smear analysis of clinical samples was performed and provided by the Medizinische Klinik 5, Hämatologie and Internistische Onkologie, Erlangen, Germany, following standard procedures. Reference measurements for healthy samples were performed using an ADVIA2120 hematology analyzer.

*Activation Measurements of Leukocytes*: Potential activation of lymphocytes, monocytes, and neutrophils due to isolation or measurement procedures was investigated by expression of CD71, CD54, and CD64.^56^, ^57^, ^58^ Leukocytes were isolated by selective hypotonic water lysis of erythrocytes as described and fluorescently labeled with anti‐CD54‐PE, anti‐CD64‐FITC, and anti‐CD71‐FITC directly after isolation and after DH measurement. FACS measurements were performed using a BD Acurri C6 flow cytometer. Three replicates were performed for each measurement.

*Analysis of Blinded Samples*: Blinded clinical samples were obtained from the Medizinische Klinik 5, Hämatologie and Internistische Onkologie, Erlangen, Germany, without any diagnostic information. All human specimens were collected with informed consent and procedures approved by application 194_15B (clinical samples) of the Ethikkommission der Universität Erlangen.

*Data Availability*: The data that support the findings of this study are available from Siemens Healthcare GmbH (HC SI TC DBS DE) but restrictions apply to the availability of these data, which were used under license for the current study, and so are not publicly available. Data are however available from the authors upon reasonable request and with permission of Siemens Healthcare GmbH (HC SI TC DBS DE).

## Conflict of Interest

The authors declare no conflict of interest.

## Supporting information

Supplementary
