# Supplementary material for: Label‐Free High‐Throughput Leukemia Detection by Holographic Microscopy
Source: Adv Sci (Weinh). 2018 Oct 11;5(12):1800761. doi: 10.1002/advs.201800761 (PMC6299719; doi:10.1002/advs.201800761)
Supplement: Supplementary file 1 — Supplementary [file ADVS-5-1800761-s001.pdf]

Copyright WILEY-VCH Verlag GmbH & Co. KGaA, 69469 Weinheim, Germany, 2018.

ADVANCED SCIENCE
Open Access

# Supporting Information

for *Adv. Sci.,* DOI: 10.1002/advs.201800761

# Label-Free High-Throughput Leukemia Detection by Holographic Microscopy

*Matthias Ugele,* Markus Weniger, Manfred Stanzel, Michael Bassler, Stefan W. Krause, Oliver Friedrich, Oliver Hayden, and Lukas Richter**

Copyright WILEY-VCH Verlag GmbH & Co. KGaA, 69469 Weinheim, Germany, 2016.

# Supporting Information

## Label-free high-throughput leukemia detection by holographic microscopy

Matthias Ugele*, Markus Weniger, Manfred Stanzel, Michael Bassler, Stefan W. Krause, Oliver Friedrich, Oliver Hayden & Lukas Richter

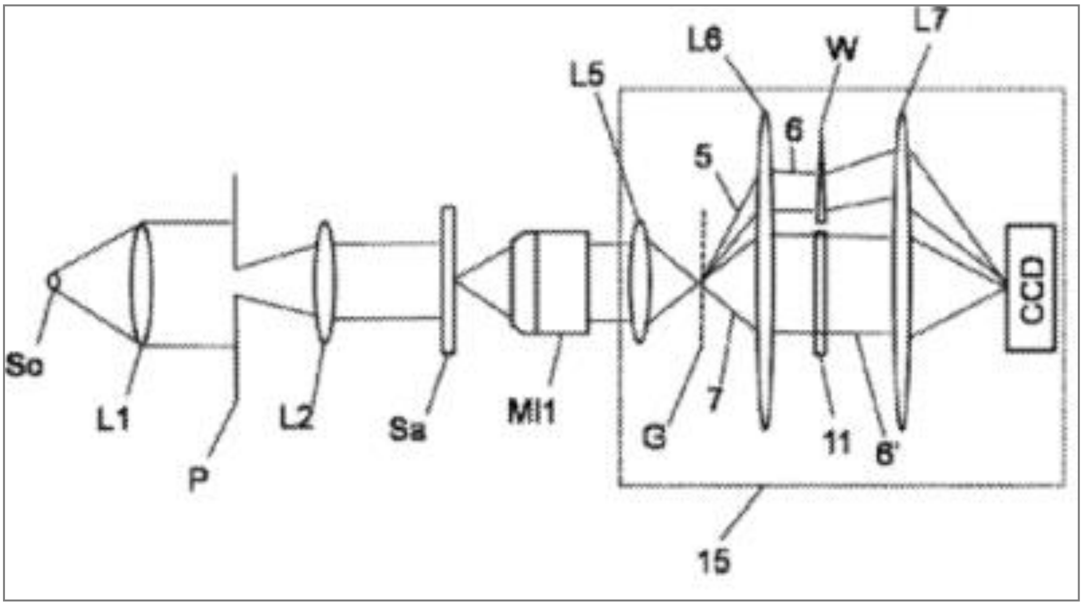


**Figure S1.** Experimental setup of differential DH. The light beam emitted by a partially coherent 528 nm SLED first passes the sample, which is located in the back focal plane of the microscope objective. The light beam is then split by a diffraction grating into a diffracted beam (reference) and a non-diffracted beam (object beam). The diffraction beam is then recombined with the object beam and focused on an imaging device. A detailed description of the microscopic setup and working principle is described in references [41, 42]. G, Grating; L1, L2, L5, L6 and L7, lenses; M11, microscope objective; P, pinhole; Sa, sample; So, Illumination source (528 nm SLED); W, Wedge; 5, Diffracted light beam; 6, Non-zero order diffracted parallel light beam; 7, Non-diffracted light beam; 15, Interferometer. Reproduced with permission. [42]

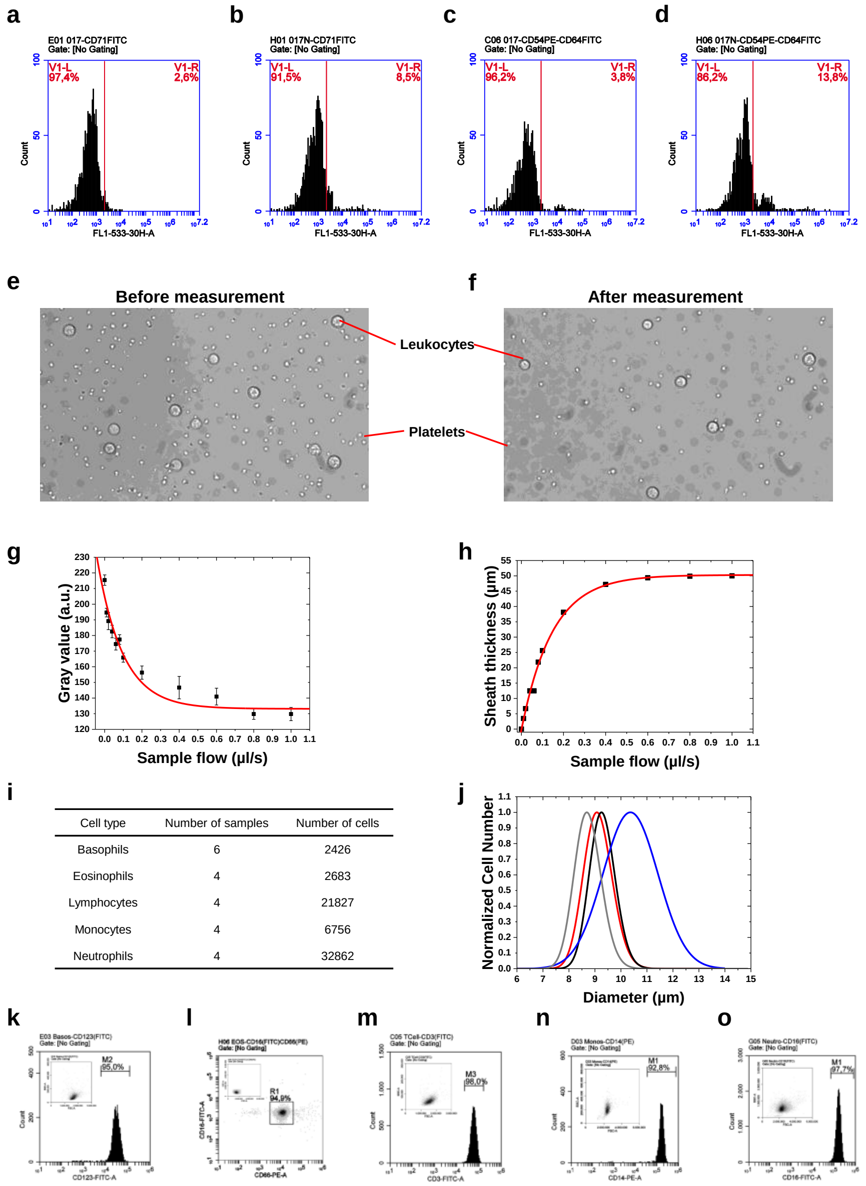


| Cell type | Number of samples | Number of cells |
|---|---|---|
| Basophils | 6 | 2426 |
| Eosinophils | 4 | 2683 |
| Lymphocytes | 4 | 21827 |
| Monocytes | 4 | 6756 |
| Neutrophils | 4 | 32862 |

**Figure S2.** Influence of sample preparation on leukocytes, analysis of sample stream height and characteristics of purified leukocyte populations used for SVM and PCA. a-b) Activation measurements of lymphocytes directly after isolation (a) and directly after DHM measurement (b). Leukocytes were fluorescently labeled with anti-CD71-FITC. FACS measurements were performed using a BD Acurri C6 flow cytometer. Each plot shows representative measurements from one sample. c-d) Activation measurements of monocytes and neutrophils directly after isolation (c) and directly after DHM measurement (d). Leukocytes were fluorescently labeled with anti-CD54-PE and anti-CD64-FITC. FACS measurements were performed using a BD Acurri C6 flow cytometer. Each plot shows

representative measurements from one sample. e-f) Phase contrast images of leukocytes isolated from whole blood by selective lysis and depletion of erythrocytes before (e) and directly after measurement (f; time from measurement to imaging < 10 minutes). No activated or apoptotic cells are present before and after measurement indicating that leukocytes are neither negatively influenced by sample preparation nor microfluidic forces used for establishment of a single cell layer. Phase contrast images were acquired using a Baumer HXG20 camera mounted on a Leica DM 2500 M. g) Determination of sample stream height using methylene blue. 0.1 M methylene blue in autoMACS® Rinsing Solution (Milteny Bitoec) was used to examine the sample stream height at different sample flow conditions (1 - 0 µl/s) using a Leica DM 2500 M microscope with a Baumer HXG20 camera. Two-dimensional-sheath flow conditions remained constant (see Methods section). The grey value for each sample flow condition was measured at three different positions inside the channel using ImageJ. For each flow conditions, three measurements were performed. The mean values ± standard deviation are displayed. h) Calculated sample sheath thickness in µm corresponding to measured grey values shown in g. i) Number of samples and total number of cells of purified basophils, eosinophils, lymphocytes, monocytes and neutrophils used for classification by SVM and PCA. For basophils, six different samples were used because of the resulting low cell numbers after purification. j) Normalized diameter distributions of purified monocytes from the four different samples listed in i measured by a Beckman Coulter Z2. Sample 4 (blue) shows a significantly broader diameter distribution than the other three samples which explains the separation of monocytes into two populations visible in Fig. 1d. k-o) Purity of enriched basophils, eosinophils, T-cells, monocytes and neutrophils labeled with anti-CD123-FITC, anti-CD16-FITC and anti-CD66-PE, anti-CD3-FITC, anti-CD14-PE and anti-CD16-FITC, respectively. FACS measurements were performed using a BD Accuri C6 flow cytometer. Insets show forward vs. side scatter plots of ungated populations. Each plot shows representative measurement from one sample.

**a**

| | Confusion matrix | | | | |
|---|---|---|---|---|---|
| | Basophils | Eosinophils | Lymphocytes | Monocytes | Neutrophils |
| Basophils | 80.36 | 0.60 | 10.54 | 4.97 | 0.41 |
| Eosinophils | 0.83 | 88.96 | 0.05 | 0.18 | 2.30 |
| Lymphocytes | 13.70 | 2.69 | 88.87 | 2.37 | 0.39 |
| Monocytes | 4.46 | 2.39 | 0.46 | 91.42 | 2.06 |
| Neutrophils | 0.66 | 5.37 | 0.07 | 1.07 | 94.84 |
| | Overall statistics | | | | |
| Accuracy | 0.92 | | | | |
| Kappa | 0.87 | | | | |
| | Statistics by class | | | | |
| Class | Basophils | Eosinophils | Lymphocytes | Monocytes | Neutrophils |
| Sensitivity | 0.80 | 0.88 | 0.89 | 0.91 | 0.95 |
| Specificity | 0.96 | 0.98 | 0.98 | 0.98 | 0.99 |

**b**

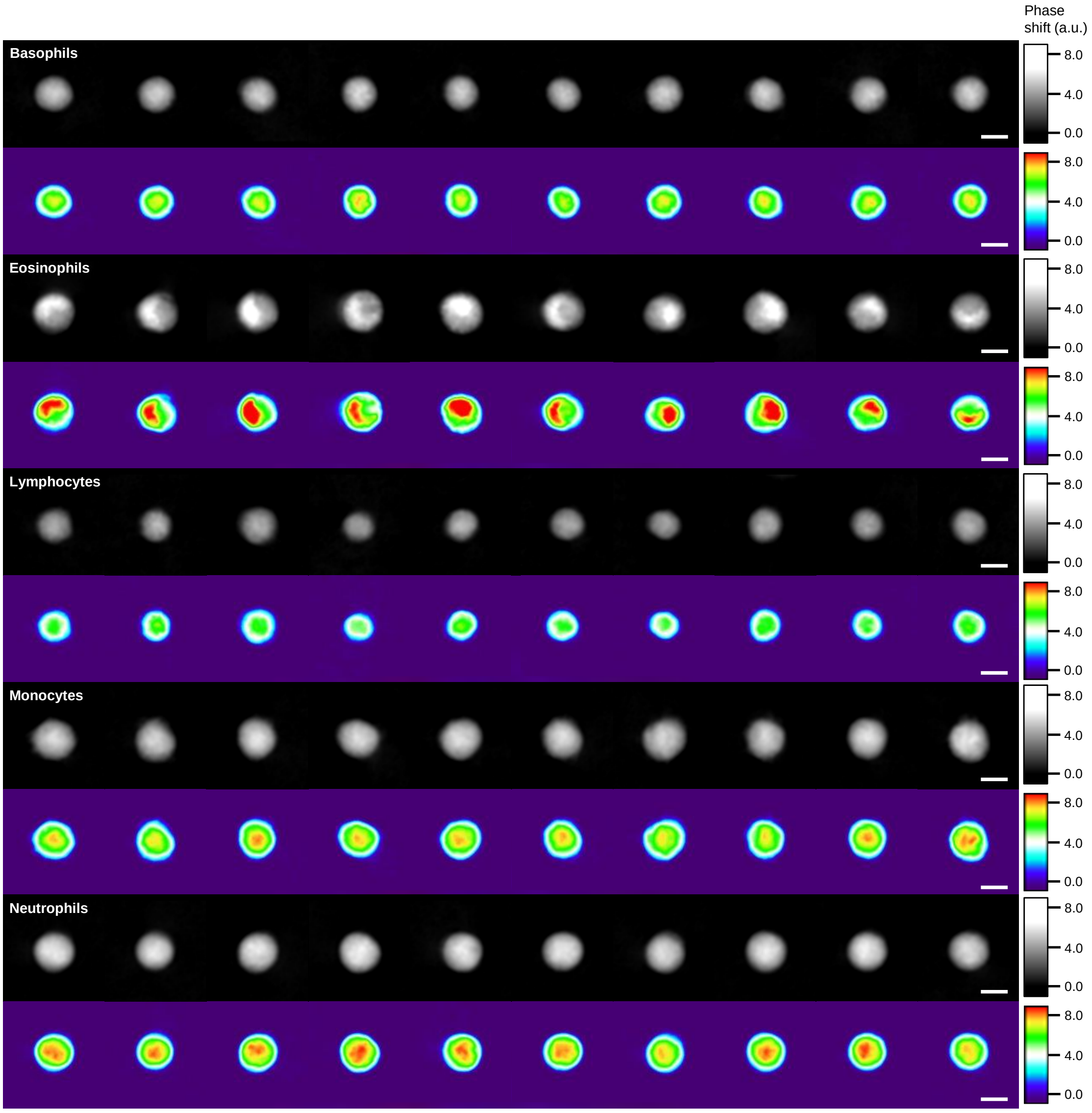


**Figure S3.** Statistics for five-part differential analysis by support vector machine (SVM) and multiple phase images of leukocyte subtypes. a) Confusion matrix and statistics for five-part

differential analysis by SVM. Accuracy: agreement between observed and predicted classes. Kappa statistics: this statistics accounts for the class distribution of the training set samples. Kappa takes values between -1 and 1, with value 0 for no agreement between observed and predicted classes, and value 1 for perfect model predictions. Sensitivity: the rate that the event is predicted correctly for all samples having the event. Sensitivity = number of samples with the event and predicted to have the event / number of samples having the event. Specificity: the rate that nonevent samples are predicted as nonevents. Specificity = number of samples without the event and predicted as nonevents / number of samples without the event. b) Reconstructed phase images and their corresponding heat maps of purified basophils, eosinophils, lymphocytes, monocytes and neutrophils from multiple healthy donors. For each cell type, ten different cells were selected randomly from the respective populations shown in Figure 1d, e. The observed conformities within each population indicate that the random orientation of the cell nuclei did not significantly affect cell morphology and thus, did not affect the subtyping. Heat maps were obtained using the ImageJ ‘HeatMap Histogram’ plugin.

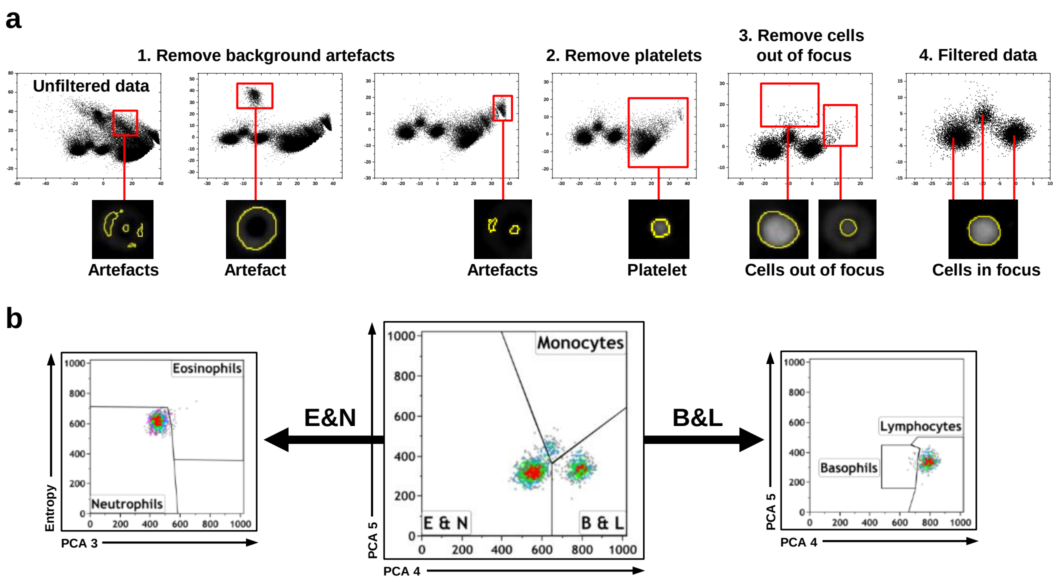


c

| Cell type | Neutrophils | Lymphocytes | Monocytes | Eosinophils | Basophils |
|---|---|---|---|---|---|
| Mean day 1 | 50.1 ± 0.6 | 36.7 ± 0.7 | 8.4 ± 0.2 | 2.3 ± 0.3 | 1.9 ± 0.7 |
| Mean day 2 | 49.8 ± 0.8 | 35.9 ± 0.9 | 9.7 ± 0.8 | 2.1 ± 0.3 | 2.6 ± 0.4 |
| Mean day 3 | 51.7 ± 2.2 | 35.2 ± 1.6 | 8.4 ± 0.7 | 2.6 ± 0.2 | 2.1 ± 0.1 |
| Mean day 1-3 | 50.7 ± 0.9 | 35.9 ± 0.8 | 8.8 ± 0.7 | 2.3 ± 0.2 | 2.2 ± 0.3 |
| Mean ADVIA day 1-3 | 50.2 ± 0.6 | 37.1 ± 0.7 | 8.4 ± 0.4 | 3.3 ± 0.7 | 0.8 ± 0.2 |

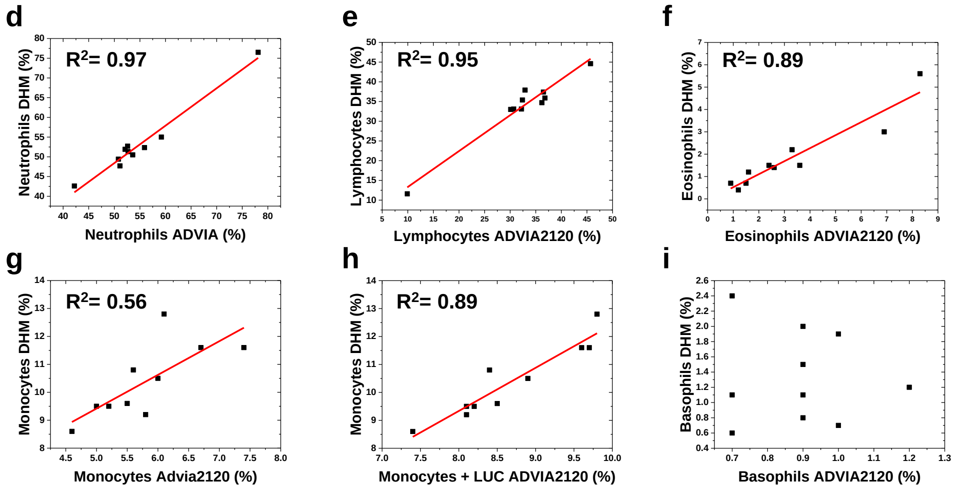


**Figure S4.** Gating strategy for label-free five-part differential of healthy samples and stability of DHM differential measurements. a) Prior to analysis, background artefacts, platelets and cells out-of-focus were removed. Background artefacts were removed by thresholding of the parameters radius variance, aspect ratio, circularity, sphericity, cell area and optical height minimum. Platelets were removed by thresholding of the parameter cell area. Cells out-of-focus were removed by thresholding of the parameters biconcavity, equivalent diameter, optical height maximum, solidity, contrast, and mass center shift. Parameters PCA 4 (x-axis)

and PCA 5 (y-axis) are plotted. b) Gating strategy for label-free five-part differential of healthy samples. Single cell data of healthy donors were first divided in the sections B&L (basophils and lymphocytes), monocytes and E&N (eosinophils and neutrophils). Basophils and lymphocytes, respectively eosinophils and neutrophils, were further separated using a combination of the parameters PCA4/PCA5 and PCA3/entropy. Density plots show representative single cell data from one sample. c) Five-part differential measurements of same donor at three following days illustrating the stability of DHM measurements. The mean of three replicates ± standard deviation is displayed for DHM measurements at day1 - 3. The monocyte and LUC (large unstained cells) populations of the ADVIA®2120 were merged for accurate comparability of DHM and ADVIA®2120. d-i) Comparison of DHM and ADVIA®2120 differential measurements by cell type of ten healthy samples. Neutrophils ($R^2$ = 0.97, Pearson R = 0.98, d), lymphocytes ($R^2$ = 0.95, Pearson R = 0.97, e) and eosinophils ($R^2$ = 0.89, Pearson R = 0.94, f) showed high correlation with the corresponding ADVIA®2120 measurements. The correlation of monocytes increased from $R^2$ = 0.56 (Pearson R = 0.75, g) to $R^2$ = 0.89 (Pearson R = 0.95, h) by merging the monocyte and LUC populations of the ADVIA®2120. Basophils showed no correlation (i).

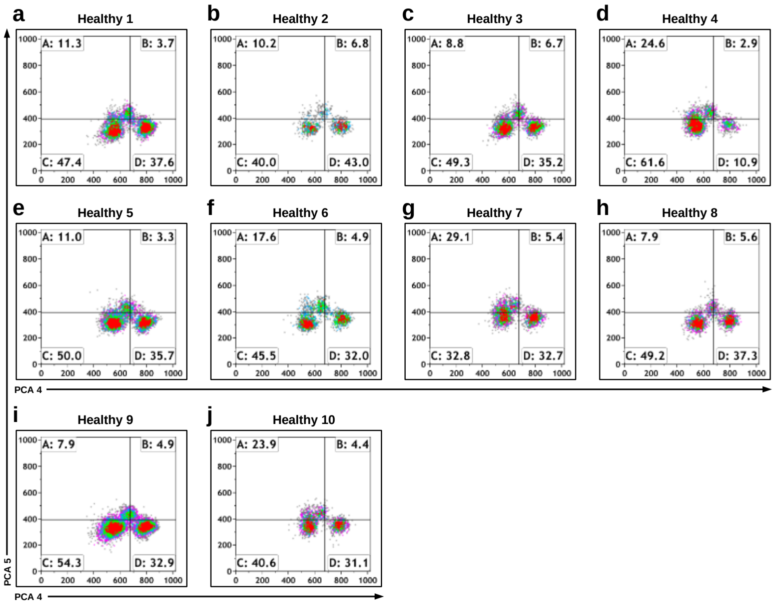


**k**

| Gate | A | B | C | D | Ly | Ne |
|---|---|---|---|---|---|---|
| Normal range | 7 - 30 | 2 - 7 | 32 - 62 | 10 - 43 | 93 - 98 | 85 - 98 |
| AML 1 | 8.9 | 50.9 | 0.9 | 39.3 | 75.7 | 12.3 |
| AML 2 | 10.7 | 75.9 | 0.4 | 13.0 | 52.9 | 1.4 |
| AML 3 | 10.5 | 79.3 | 0.6 | 9.6 | 53.3 | 3.0 |
| AML 4 | 23.0 | 31.1 | 23.2 | 22.7 | 64.9 | 73.9 |
| AML 5 | 68.5 | 10.2 | 11.6 | 9.7 | 64.2 | 4.0 |
| AML 6 | 40.9 | 11.7 | 9.6 | 37.8 | 88.0 | 26.6 |
| AML 7 | 29.0 | 27.0 | 14.8 | 29.2 | 91.8 | 42.5 |
| AML 8 | 65.2 | 7.4 | 9.8 | 17.6 | 92.9 | 28.8 |
| AML 9 | 35.1 | 54.1 | 0.3 | 10.5 | 93.1 | 70.7 |
| AML 10 | 57.9 | 22.1 | 1.9 | 18.1 | 87.0 | 8.9 |
| ALL | 15.4 | 40.2 | 7.0 | 37.4 | 71.7 | 45.2 |
| CLL 1 | 2.7 | 0.2 | 2.1 | 95.0 | 90.6 | 71.4 |
| CLL 2 | 1.5 | 16.0 | 0.8 | 81.7 | 86.6 | 59.5 |
| MPN 1 - CML | 33.9 | 3.4 | 57.4 | 5.3 | 97.1 | 80.5 |
| MPN 2 - CMML | 30.6 | 1.1 | 61.8 | 6.5 | 97.2 | 84,2 |
| MPN 3 - OMF | 27.1 | 14.9 | 35.1 | 22.9 | 89.3 | 73.2 |
| MPN 4 - OMF | 30.8 | 8.8 | 43.7 | 16.7 | 92.1 | 81.4 |
| MPN 5 - PV | 40.5 | 12.2 | 30.6 | 16.7 | 93.1 | 65.4 |
| MPN 6 - NOS | 47.6 | 6.4 | 25.5 | 20.5 | 96.6 | 54.9 |
| MPN 7 - post ET | 40.1 | 23.5 | 26.0 | 10.4 | 64.2 | 58.2 |

Abnormal
Normal range

**Figure S5.** Overview of analyzed healthy samples and differentiation of healthy and leukemic samples. a-j) Label-free ungated density plots of analyzed healthy samples. Parameters PCA4 and PCA5 are plotted. Percentages of cells are indicated for each plot quadrant. k) Percentage distribution of analyzed clinical samples in the quadrants A, B, C and D and in the gates Ly (basophils and lymphocytes) and Ne (eosinophils and neutrophils). Deviations from the healthy ranges are indicated by red markings. Samples were classified as leukemic when they deviated from the healthy range in at least one quadrant, which is the case for all analyzed clinical samples.

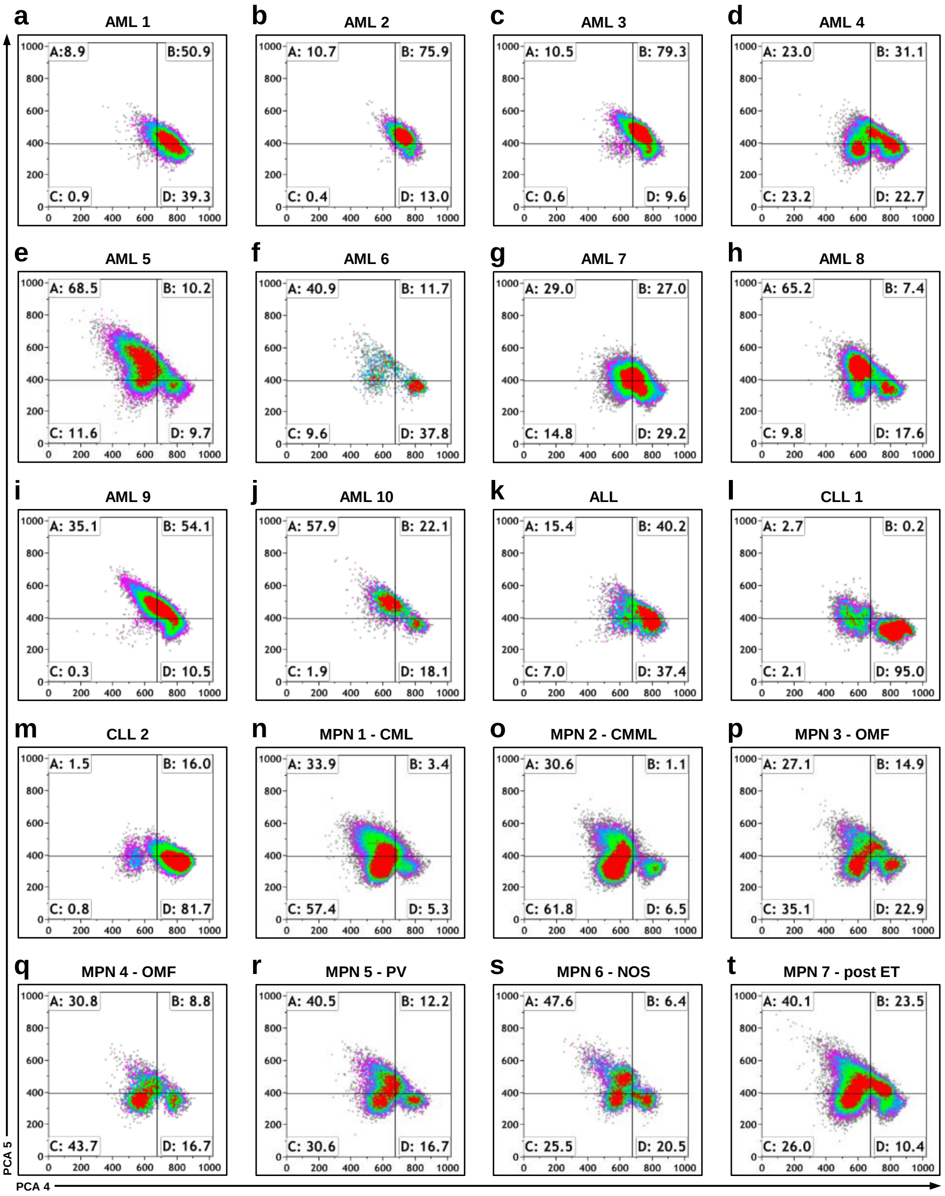


**Figure S6.** Overview of label-free, ungated single cell data of analyzed clinical samples. a-j) Label-free, ungated density plots of AML samples. k) Label-free, ungated density plot of ALL sample. l, m) Label-free, ungated density plots of CLL samples. n-t) Label-free, ungated density plots of MPN samples including CML (n), CMML (o), OMF (p, q), PV (r), MPN NOS (s) and post ET (t) . Parameters PCA 4 and PCA 5 are plotted. Percentages of cells are indicated for each plot quadrant.

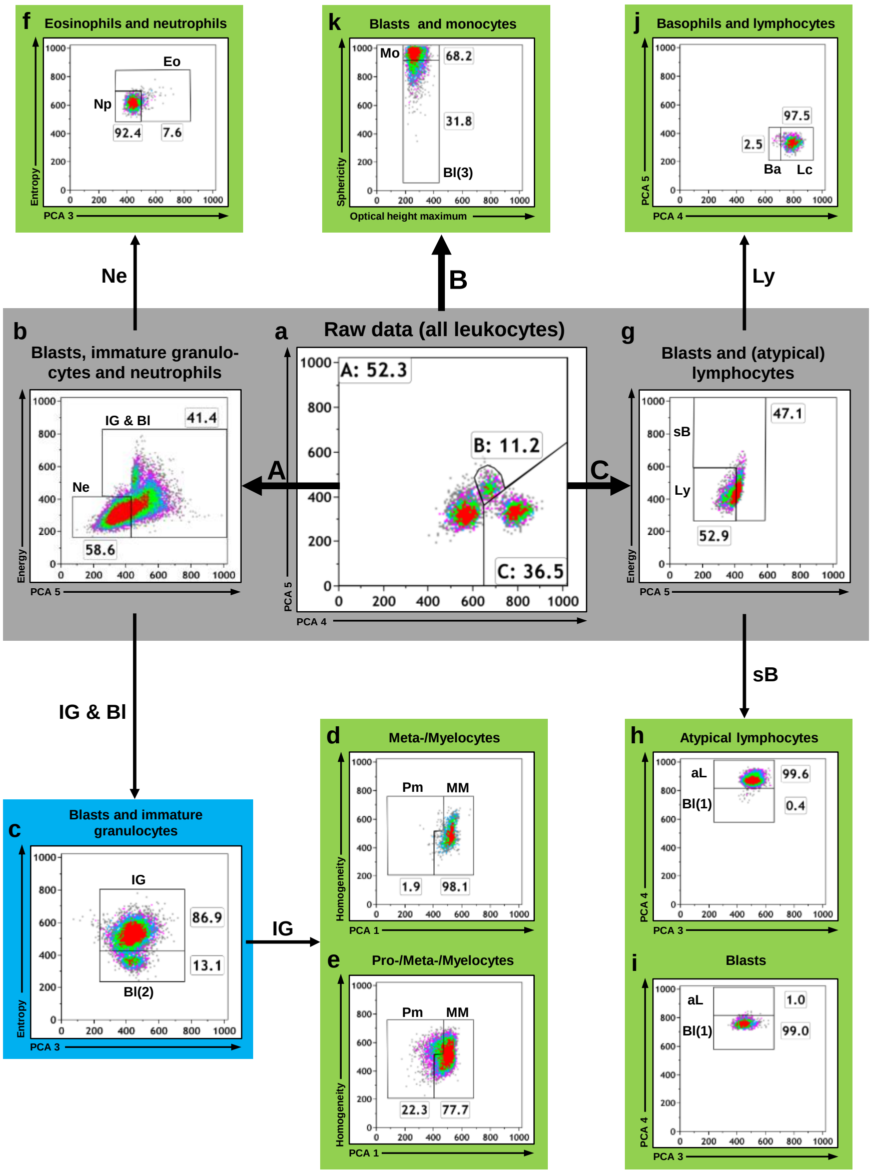


**Figure S7.** Gating strategy for differentiation of nine leukocyte subtypes. a) Single cell data of healthy sample divided in three sections A, B and C. Section A contains eosinophils, neutrophils and potentially blasts and immature granulocytes. Section B contains monocytes and potentially blasts. Section C contains basophils, lymphocytes, and potentially blasts, and atypical lymphocytes. Parameters PCA4 and PCA5 are plotted. b) Section A from sample MPN7 plotted with parameters PCA5 and energy. In addition to eosinophils and neutrophils (gate Ne), a high amount of immature granulocytes respectively blasts is present (gate IG & Bl). c) Gate IG & Bl from sample shown in b plotted with parameters PCA3 and entropy. Blasts (Bl(2)) and immature granulocytes (IG) are separated. d) Gate IG from sample AML4

plotted with parameters PCA1 and homogeneity. In agreement with blood smear analysis only meta-/myelocytes are present in this sample (Table S2). Metamyelocytes and myelocytes could not be further separated with the received samples. e) Gate IG from sample shown in c plotted with parameters PCA1 and homogeneity. In agreement with blood smear analysis meta-/myelocytes and promyelocytes are present. f) Gate Ne from healthy sample plotted with parameters PCA3 and entropy. Eosinophils (Eo) and neutrophils (Np) are separated. g) Section C from sample AML2 plotted with parameters PCA5 and energy. In addition to basophils and lymphocytes (gate Ly), a high amount of suspected blasts is present (gate sB). h) Gate sB from sample CLL1 plotted with parameters PCA3 and PCA4. Exclusively atypical lymphocytes (aL) are present. i) Gate sB from sample AML2 plotted with parameters PCA3 and PCA4. Exclusively blasts (Bl(1)) are present. j) Gate Ly from healthy sample plotted with parameters PCA4 and PCA5. Basophils (Ba) and lymphocytes (Lc) are separated. k) Section C from sample AML2 plotted with parameters optical height maximum and sphericity. Blasts (Bl(3)) and monocytes are separated. Each density plot shows representative single cell data from one patient. Percentages of cells are indicated for each gate or plot quadrant. Five-part differential data of ten healthy donors and blood smear analysis of 20 leukemic samples were used as reference. For a detailed description of plotted parameters see Supplemental Table 1.

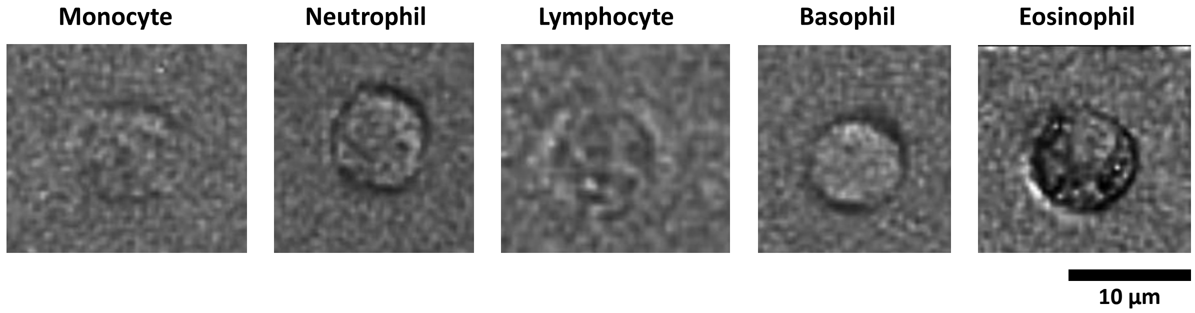


**Figure S8.** Intensity images of different leukocyte subtypes. Reconstructed intensity images of purified basophils, eosinophils, lymphocytes, monocytes and neutrophils from healthy donors. Leukocytes do not show distinct morphological differences from each other, except eosinophils.

**Table S1.** List, description and calculation of morphological parameters.

| Parameter | Unit | Description |
|---|---|---|
| Cell Area | µm² | Area contained by cell contour |
| Perimeter | µm | Cell contour perimeter |
| Width | µm | Width of rotated bounding rectangle with minimum area |
| Height | µm | Height of rotated bounding rectangle with minimum area |
| Aspect ratio | a. u. | max (Width,Height) /min (Width,Height) |
| Circularity | a. u. (0 - 1) | The circularity of the cell contour. Calculated by 4π* cellArea / perimeter². Circularity of a perfect circle is 1. |
| Radius mean | µm | The mean distance between the centroid of the contour and each contour support point |
| Radius variance | a. u. | The variance of the distance between the centroid of the contour and each contour support point |
| Solidity | a. u. | Ratio of contour area to its convex hull area |
| Equivalent Diameter | µm | The diameter of the circle whose area is same as the contour area |
| Optical volume | µm³ | The overall optical volume is the sum of all pixel volumes inside the contour |
| Optical height maximum | a. u. | The maximum phase value inside the contour |
| Optical height minimum | a. u. | The minimum phase value inside the contour |
| Optical height mean | a. u. | The mean phase value inside the contour |
| Optical height variance | a. u. | The phase value variance inside the contour |
| Biconcavity | a. u. (-1 - 1) | A measurement for a biconcave shape of the cell. Describes the correlation of phase values on a horizontal and vertical cut in the middle of the cell contour to an idealized biconcave cell modelled by $-4x^4 + 4x^2 + 0{,}5$ is calculated. |
| Sphericity | a. u. (-1 - 1) | A measurement for a spherical shape of the cell. Describes the correlation of phase values on a horizontal and vertical cut in the middle of the cell contour to an idealized spherical cell modelled by $-x^2 + 1$ is calculated. |
| Mass Center shift | a. u. | Euclidian distance between geometric centroid and mass centroid |
| Contrast | a. u. | Intensity contrast between a pixel and its neighbor based on GLCM of phase values converted to 6bit grayscale image for the pixels inside cell contour. |
| Dissimilarity | a. u. | Dissimilarity measure based on GLCM of phase values converted to 6bit grayscale image for the pixels inside cell contour. |
| Homogenity | a. u. | Homogenity measure based on GLCM of phase values converted to 6bit grayscale image for the pixels inside cell contour. |
| Energy | a. u. | Energy measure (sum of squared elements) based on GLCM of phase values converted to 6bit grayscale image for the pixels inside cell contour. |
| Entropy | a. u. | Entropy measure based on GLCM of phase values converted to 6bit grayscale image for the pixels inside cell contour. |
| PCA 1 | a. u. | PCA principle axis 1 calculated with complete 5 part differential training data. |
| PCA 2 | a. u. | PCA principle axis 2 calculated with complete 5 part differential training data. |
| PCA 3 | a. u. | PCA principle axis 3 calculated with complete 5 part differential training data. |
| PCA 4 | a. u. | PCA principle axis 1 calculated only with basophil- and lymphocyte training data. |
| PCA 5 | a. u. | PCA principle axis 2 calculated only with basophil- and lymphocyte training data. |
| PCA 6 | a. u. | PCA principle axis 3 calculated only with basophil- and lymphocyte training data. |

**Table S2.** List, diagnosis and blood smear differential count of leukemic samples.

| | | | Blood smear differential count (%) | | | | | | | | | | |
|---|---|---|---|---|---|---|---|---|---|---|---|---|---|
| Sample | Diagnosis | WBC *$10^3$/µl | N | L | M | E | B | PM | MM | MY | Bl | aL | Smudge cells |
| AML1 | AML | 17.4 | - | 17 | 1 | - | - | - | - | - | 82 | - | - |
| AML2 | AML | 5.7 | - | 11 | 2 | - | - | - | - | - | 87 | - | - |
| AML3 | AML | 17.8 | 2 | 11 | 1 | - | - | - | - | - | 86 | - | - |
| AML4 | AML | 26.5 | 42 | 22 | 9 | 2 | - | - | 2 | - | 23 | - | - |
| AML5 | AML | 14.0 | 44 | 10 | - | - | - | 8 | 5 | 8 | 25 | - | - |
| AML6 | AML | 1.1 | 31 | 37 | 3 | 7 | 1 | - | - | - | 21 | - | - |
| AML7 | AML | 83.2 | 22 | 13 | 5 | 3 | 13 | 1 | 1 | 1 | 41 | - | - |
| AML8 | AML, mm | 32.7 | 3 | 16 | 74 | - | 1 | - | - | - | 6 | - | - |
| AML9 | AML | 35.5 | 1 | 12 | - | - | - | - | - | - | 87 | - | - |
| AML10 | AML | 3.4 | 7 | 19 | - | - | - | - | 2 | 4 | 68 | - | - |
| ALL | T-ALL | 10.3 | 22 | 15 | 5 | 1 | - | - | - | - | 57 | - | - |
| CLL1 | CLL | 31.4 | 8 | 76 | 1 | - | - | - | - | 1 | - | - | 14 |
| CLL2 | CLL | 202 | 2 | 92 | 1 | 1 | - | - | - | - | - | - | 4 |
| MPN1 | CML | 197.9 | 55 | 4 | 2 | 2 | 3 | 5 | 12 | 13 | 4 | - | - |
| MPN2 | CMML | 122.0 | 51 | 13 | 7 | - | - | 5 | 14 | 4 | 6 | - | - |
| MPN3 | OMF | 8.7 | 49 | 21 | 5 | 4 | 6 | 3 | 4 | 3 | 5 | - | - |
| MPN4 | OMF | 4.3 | 51 | 28 | 2 | - | - | 0 | 9 | 8 | - | 2 | - |
| MPN5 | PV | 5.6 | 43 | 17 | 5 | - | 2 | 2 | 4 | 9 | 18 | - | - |
| MPN6 | MPN NOS | 10.4 | 37 | 17 | 18 | 3 | 14 | 2 | 5 | 3 | 1 | - | - |
| MPN7 | post ET | 29.6 | 35 | 10 | 2 | 9 | - | 2 | 5 | 9 | 28 | - | - |
| AML8 Remission | Remission | 3.9 | 47 | 25 | 10 | 5 | 4 | 2 | 3 | 4 | - | - | - |

AML, acute myeloid leukemia; ALL, acute lymphocytic leukemia, CLL, chronic lymphocytic leukemia; MPN, myeloproliferative neoplasm; CML, chronic myelogenous leukemia; CMML, chronic myelomonocytic leukemia; OMF, osteomyelofibrosis; PV, polycythemia vera; NOS, not otherwise specified; ET, essential thrombocythemia; mm, myelomonocytic; N, neutrophils; L, lymphocytes; M, monocytes; E, eosinophils; B, basophils; PM, promyelocytes; MM, metamyelocytes; MY, myelocytes; Bl, Blasts; aL, atypical lymphocytes.

**Table S3.** DH nine-part differential count of analyzed healthy and pathological samples.

| | | | DHM differential count (%) | | | | | | | | | |
|---|---|---|---|---|---|---|---|---|---|---|---|---|
| Sample | Diagnosis | Number of cells | N | L | M | E | B | IG (all) | PM | MM/ MY | Bl | aL |
| Healthy 1 | - | 5,564 | 42.7 | 36.9 | 8.6 | 7.3 | 1.3 | 1.7 | 0.0 | 1.7 | 0.8 | 0.7 |
| Healthy 2 | - | 1,144 | 37.9 | 43.8 | 9.8 | 3.9 | 0.4 | 2.6 | 0.0 | 2.6 | 0.9 | 0.7 |
| Healthy 3 | - | 3,979 | 47.0 | 34.7 | 10.8 | 3.9 | 0.9 | 1.2 | 0.0 | 1.2 | 0.9 | 0.6 |
| Healthy 4 | - | 3,350 | 68.9 | 11.8 | 9.7 | 2.9 | 0.3 | 5.3 | 0.1 | 5.2 | 0.9 | 0.2 |
| Healthy 5 | - | 5,779 | 45.9 | 34.3 | 8.6 | 6.3 | 1.9 | 1.6 | 0.0 | 1.6 | 0.8 | 0.6 |
| Healthy 6 | - | 2,318 | 45.3 | 31.9 | 11.8 | 3.8 | 0.8 | 4.2 | 0.0 | 4.2 | 1.5 | 0.7 |
| Healthy 7 | - | 2,945 | 40.8 | 33.9 | 8.6 | 5.1 | 0.5 | 8.1 | 0.1 | 8 | 1.7 | 1.3 |
| Healthy 8 | - | 2,878 | 41.2 | 37 | 8.5 | 8.9 | 1.3 | 0.8 | 0.0 | 0.8 | 1.6 | 0.7 |
| Healthy 9 | - | 11,150 | 44.4 | 32.5 | 8.1 | 11.3 | 1.1 | 1.1 | 0.0 | 1.1 | 0.7 | 0.8 |
| Healthy 10 | - | 2,561 | 46.8 | 31.9 | 9.6 | 2.9 | 0.6 | 6.4 | 0.0 | 6.4 | 1.1 | 0.7 |
| AML1 | AML | 18,975 | 0.6 | 46.6 | 15.1 | 0.2 | 2.9 | 1.9 | 0.1 | 1.8 | 31.6 | 1.1 |
| AML2 | AML | 9,578 | 0.1 | 18.6 | 34.2 | 0.0 | 1.5 | 1.6 | 0.1 | 1.5 | 43.8 | 0.2 |
| AML3 | AML | 35,263 | 0.6 | 12.5 | 33.3 | 0.1 | 0.3 | 2.5 | 0.2 | 2.3 | 50.6 | 0.1 |
| AML4 | AML | 25,084 | 21.3 | 24 | 19.6 | 5.3 | 2.0 | 6.5 | 0.1 | 6.4 | 15.9 | 5.4 |
| AML5 | AML | 29,162 | 15.6 | 10 | 15.1 | 2.1 | 1.3 | 43.6 | 15.8 | 27.8 | 11.9 | 0.4 |
| AML6 | AML | 1,909 | 14.9 | 40.9 | 8.9 | 3.8 | 0.7 | 23.9 | 4.8 | 19.1 | 6 | 0.9 |
| AML7 | AML | 56,219 | 10.1 | 23 | 27.4 | 3.0 | 13 | 4.4 | 0.1 | 4.3 | 18.7 | 0.4 |
| AML8 | AML, mm | 34,379 | 13.6 | 17.9 | 22.9 | 1.5 | 1.3 | 37.1 | 0.9 | 36.2 | 5.3 | 0.4 |
| AML9 | AML | 84,638 | 0.4 | 14.2 | 52.1 | 0.1 | 0.5 | 8.4 | 0.8 | 7.6 | 24.2 | 0.1 |
| AML10 | AML | 5,259 | 2.9 | 19.4 | 32.2 | 0.3 | 0.8 | 29.5 | 2.8 | 26.7 | 14.3 | 0.6 |
| ALL | T-ALL | 9,845 | 7.0 | 43.7 | 15.4 | 0.8 | 2.8 | 5.4 | 0.6 | 4.8 | 21.6 | 3.3 |
| CLL1 | CLL | 106,670 | 2.6 | 86.2 | 0.7 | 0.2 | 0.3 | 1.1 | 0.1 | 1.0 | 0.1 | 8.8 |
| CLL2 | CLL | 90,716 | 0.9 | 80.1 | 3.8 | 0.2 | 1.3 | 0.3 | 0.0 | 0.3 | 4.8 | 8.6 |
| MPN1 | CML | 64,232 | 55.8 | 3.4 | 10.8 | 6.2 | 4.3 | 14.9 | 3.0 | 11.9 | 4.6 | 0.0 |
| MPN2 | CMML | 46,245 | 65.1 | 6.1 | 8.6 | 4.6 | 1.0 | 12.6 | 2.2 | 10.4 | 1.7 | 0.3 |
| MPN3 | OMF | 17,481 | 30.3 | 22.1 | 12.1 | 7.2 | 2.8 | 12.1 | 2.6 | 9.5 | 12.4 | 1.0 |
| MPN4 | OMF | 7,257 | 44.1 | 17.5 | 12.5 | 5.9 | 1.7 | 11.2 | 1.7 | 9.5 | 6.7 | 0.4 |
| MPN5 | PV | 13,073 | 33.2 | 16.1 | 18.7 | 2.0 | 2.9 | 17.5 | 3.4 | 14.1 | 9.1 | 0.5 |
| MPN6 | MPN NOS | 9,842 | 28.8 | 19.7 | 17.3 | 2.3 | 3.6 | 24.8 | 6.0 | 18.8 | 3.4 | 0.1 |
| MPN7 | post ET | 43,107 | 25.9 | 13.9 | 12.7 | 8.0 | 0.8 | 21.1 | 4.9 | 16.2 | 17.2 | 0.4 |
| AML8 Remission | Remission | 4,082 | 36.6 | 20.5 | 19.4 | 3.5 | 1.3 | 14.4 | 0.7 | 13.7 | 4.1 | 0.2 |

Percentage of neutrophils are derived from gate ‘Np’, lymphocytes from gate ‘Lc’, monocytes from gate ‘Mo’, eosinophils from gate ‘Eo’, basophils from gate ‘Ba’, immature granulocytes (all) from gate ‘IG’, promyelocytes from gate ‘Pm’, meta-/myelocytes from gate ‘MM’, blasts from the sum of the gates ‘Bl(1)’, ‘Bl(2)’ and ‘Bl(3)’, and atypical lymphocytes from the gate ‘aL’ (see Supplemental Fig. 6).

AML, acute myeloid leukemia; ALL, acute lymphocytic leukemia, CLL, chronic lymphocytic leukemia; MPN, myeloproliferative neoplasm; CML, chronic myelogenous leukemia; CMML, chronic myelomonocytic leukemia; OMF, osteomyelofibrosis; PV, polycythemia vera; NOS, not otherwise specified; ET, essential thrombocythemia; mm, myelomonocytic; N, neutrophils; L, lymphocytes; M, monocytes; E, eosinophils; B, basophils; IG, immature granulocytes; PM, promyelocytes; MM, metamyelocytes; MY, myelocytes; Bl, Blasts; aL, atypical lymphocytes.

**Table S4.** List and diagnosis of analyzed blinded clinical samples.

| Sample | DHM | Clinician |
|---|---|---|
| Blinded 1 | MPN | AML, monocytic |
| Blinded 2 | Healthy | Healthy |
| Blinded 3 | AML | AML |
| Blinded 4 | AML | AML |
| Blinded 5 | MPN | Strong left shift including promyelocytes |
| Blinded 6 | LL | CLL |
| Blinded 7 | AML | AML |
| Blinded 8 | Healthy | ALL |
| Blinded 9 | MPN | AML, monocytic |
| Blinded 10 | LL | CLL |
| Blinded 11 | MPN | Healthy (CML in remission) |
| Blinded 12 | AML | MPN with increased blast amount |
| Blinded 13 | LL | CLL |
| Blinded 14 | AML | AML |
| Blinded 15 | LL | CLL |
| Blinded 16 | MPN | MPN (CML) |
| Blinded 17 | AML | AML |
| Blinded 18 | AML | AML |
| Blinded 19 | AML | AML |
| Blinded 20 | Healthy | Healthy |

AML, acute myeloid leukemia; ALL, acute lymphocytic leukemia, CLL, chronic lymphocytic leukemia; MPN, myeloproliferative neoplasm; CML, chronic myelogenous leukemia; LL, lymphatic leukemia.
